# High-Throughput Direct Mass Spectrometry-Based Metabolomics to Characterize Metabolite Fingerprints Associated with Alzheimer’s Disease Pathogenesis

**DOI:** 10.3390/metabo8030052

**Published:** 2018-09-18

**Authors:** Raúl González-Domínguez, Ana Sayago, Ángeles Fernández-Recamales

**Affiliations:** 1 Department of Chemistry, Faculty of Experimental Sciences, University of Huelva, 21007 Huelva, Spain; ana.sayago@dqcm.uhu.es (A.S.); recamale@dqcm.uhu.es (A.F.-R.); 2 International Campus of Excellence ceiA3, University of Huelva, 21007 Huelva, Spain; 3 Biomarkers & Nutrimetabolomics Laboratory, Department of Nutrition, Food Sciences and Gastronomy, Faculty of Pharmacy and Food Sciences, University of Barcelona, 08028 Barcelona, Spain

**Keywords:** metabolomics, direct mass spectrometry, Alzheimer’s disease, pathogenesis, biomarkers

## Abstract

Direct mass spectrometry-based metabolomics has been widely employed in recent years to characterize the metabolic alterations underlying Alzheimer’s disease development and progression. This high-throughput approach presents great potential for fast and simultaneous fingerprinting of a vast number of metabolites, which can be applied to multiple biological matrices including serum/plasma, urine, cerebrospinal fluid and tissues. In this review article, we present the main advantages and drawbacks of metabolomics based on direct mass spectrometry compared with conventional analytical techniques, and provide a comprehensive revision of the literature on the use of these tools in the investigation of Alzheimer’s disease.

## 1. The Potential of Direct Mass Spectrometry-Based Metabolomics

**Figure 1:**
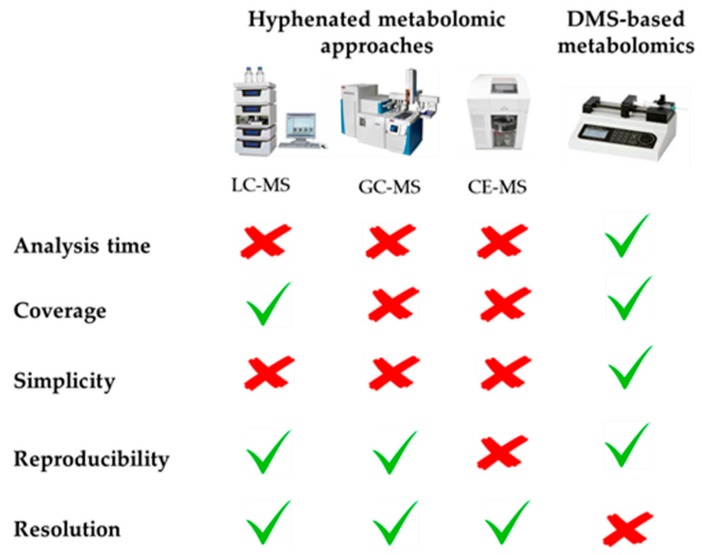
Advantages and drawbacks of DMS-based metabolomics compared with conventional hyphenated approaches.

Metabolomics requires the use of powerful and versatile analytical techniques with the aim of covering the largest number of compounds comprising the great complexity of the metabolome, which is composed of metabolites with diverse molecular weights, polarities, acid-base properties, and other physicochemical characteristics. To this end, multiple metabolomic platforms have been proposed in the literature, including nuclear magnetic resonance (NMR), and mass spectrometry (MS) coupled to liquid chromatography (LC), to gas chromatography (GC), or to capillary electrophoresis (CE), each of them having their own strengths and weaknesses. For this reason, the combination of several of these complementary techniques is becoming a powerful workhorse to accomplish a global characterization of the metabolome [1,2,3]. Among these analytical tools, direct mass spectrometry (DMS)-based metabolomics has usually been relegated to the background due to its inherent drawbacks, such as the impossibility of resolving chemical isomers and problems associated with ion suppression due to the introduction of the whole sample into the mass spectrometry system without previous chromatographic or electrophoretic separation. However, some recently published review articles have also highlighted the great potential of this metabolomic approach, as illustrated in Figure 1 [4,5,6,7]. The most notable advantage of this tool is its high-throughput screening capability, due to the absence of a previous time-consuming separation step, which considerably reduces the total analysis time, thus allowing the analysis of hundreds of samples per day. The elimination of this chromatographic/electrophoretic separation also prevents the introduction of biased and selective retention mechanisms, so that DMS enables the simultaneous measurement of a huge number of metabolites, covering a wide physicochemical space. In this sense, it should also be noted that multiple instrumental configurations are available for performing DMS-based metabolomics, which can be combined to increase the metabolome coverage. For non-targeted metabolomics, direct infusion mass spectrometry (DIMS) is the simplest approach, since it only needs a syringe pump to introduce the sample extract into the mass spectrometer. Complementarily, the sample can also be delivered by flow injection (FIMS) using a LC pump. On the other hand, the multi-dimensional mass spectrometry-based shotgun lipidomic (MDMS-SL) approach developed by Han et al. allows the direct quantitation of hundreds of individual lipid species by means of a selective ionization of certain category of lipid classes at certain MS conditions [8]. In this context, simpler targeted metabolomic platforms are the AbsoluteIDQ^TM^ kits developed by Biocrates Life Sciences AG (Innsbruck, Austria), focused on the FI-MS/MS-based quantification of multiple metabolite classes, including lipids (phospholipids, sphingolipids, acyl-carnitines, glycerolipids), amino acids, hexoses and biogenic amines [9]. In turn, most of these DMS-based configurations can be coupled with various complementary atmospheric pressure ionization sources. Electrospray ionization (ESI) is the most commonly employed source in non-targeted metabolomics, which allows the simultaneous characterization of compounds with very diverse physico-chemical properties due to its sensitivity and versatility. Complementarily, atmospheric pressure chemical ionization (APCI) and atmospheric pressure photoionization (APPI) sources can also be employed for the ionization of less polar compounds. Thus, the combination of complementary ion sources and ionization modes (i.e., positive and negative polarities), is recommended to maximize the analytical coverage. To conclude, it is also worth noting that the lack of a separation step prior to MS detection facilitates the experimental design by avoiding common challenges associated with chromatography and electrophoresis, such as column/capillary clogging and deterioration, the need for complex data processing packages to align retention/migration times, as well as the minimization of the instrumental drift along batch analysis thanks to the reduced acquisition times usually employed in these approaches.

## 2. Alzheimer’s Disease, Mild Cognitive Impairment and Animal Models

Alzheimer’s disease (AD) is the most prevalent neurodegenerative disorder worldwide in the elderly, and is primarily characterized by neuropathological alterations associated with the deposition of amyloid plaques and the formation of intra-neuronal neurofibrillary tangles. Furthermore, numerous authors have proposed that multiple other pathological processes can also play a pivotal role in the development of this disease, such as oxidative stress, abnormal mitochondrial functioning, neuroinflammatory mechanisms, impaired metal homeostasis and many others [10,11,12]. The investigation of AD etiology involves a great challenge to the scientific community due to its great complexity and the variability of clinical symptoms, its long pre-symptomatic period, and the impossibility of studying brain microscopic changes until the final stages of the disease. For these reasons, diagnosis of AD nowadays relies on the combination of various physical, neuropsychological and laboratory tests according to the clinical criteria of the National Institute of Neurological and Communicative Disorders and the Alzheimer’s Disease and Related Disorders Association (NINCDS-ADRDA) [13]. However, this diagnostic method is only effective at advanced dementia, which hinders the application of pharmacological interventions, and in addition suffers from low specificity against other dementias as demonstrated after post mortem histopathological verification [14]. Thus, the discovery of novel biomarkers for accurate diagnosis of AD is mandatory, especially for predicting the development of disease from pre-dementia phases, also called mild cognitive impairment (MCI). MCI is a heterogeneous syndrome characterized by very mild symptoms of cognitive dysfunction, and is usually considered an intermediate pre-clinical stage of Alzheimer’s disease. Although MCI has many common features with early AD, current data suggest that some MCI forms are part of the normal aging process [15]. Therefore, there is a great need to discover potential biomarkers for diagnosis and to investigate the pathological mechanisms associated with AD and MCI development and progression.

On the other hand, animal models are very useful tools for investigating the pathogenesis of AD and associated alterations in the central nervous system at different stages along the progression of disease [16], while studies in human cohorts are limited to post-mortem brain tissue, when the disease is in its final stage. Transgenic mice, obtained by the over-expression of mutated forms of human genes associated with AD such as the amyloid precursor protein (APP), presenilin 1 (PS1), presenilin 2 (PS2) or apolipoprotein E (ApoE), are the most useful models, since the neuropathology elicited by these animals is analogous to that observed in human AD, and furthermore, biochemical routes in humans and rodents are very similar [17]. The transgenic mice most commonly employed in AD research are based on the up-regulation of the APP, including the APP_Tg2576_, APP_V717F_ and CRND8, transgenic lines, which usually show amyloid deposition in hippocampus and cortex and memory deficits, but not neuronal loss. In this vein, it has been demonstrated that the co-expression of mutated PS1, and to a lesser extent PS2, accelerates amyloid deposition, thus facilitating the appearance of the characteristic AD phenotype (APP × PS1, TASTPM). Taking into account the fact that the ε4 allele of ApoE is one of the most important risk factors for AD, several knock-in mice in which this protein is expressed have been developed, which show significant cognitive and synaptic plasticity impairments. On the contrary, only a few transgenic models expressing tauopathy have been developed to date due to the lack of knowledge of genes involves in this process in AD (TAPP, 3 × Tg).

## 3. Application of Direct Mass Spectrometry-Based Metabolomics to AD Research

Considering the multifactorial nature of AD etiology, the application of holistic metabolomic approaches is emerging for the investigation of pathological hallmarks underlying this neurodegenerative disorder and for the discovery of potential diagnostic biomarkers [2,18,19]. In particular, DMS-based metabolomics has demonstrated great potential to characterize the AD metabotype in a comprehensive manner, as discussed in this section and summarized in Table 1.

Numerous non-targeted DMS-based metabolomic studies have been conducted in serum samples, which is a very useful biofluid in clinical practice for the identification of diagnostic biomarkers in a non-invasive manner. González-Domínguez et al. employed a DIMS platform based on a two-step treatment of serum samples from AD patients to obtain a holistic snapshot of metabolite alterations associated with the early development of this neurodegenerative disorder [20,21]. The most notable findings could be associated with an abnormal homeostasis of neural membrane lipids, evidenced by reduced levels of circulating phospholipids containing polyunsaturated fatty acids (PUFAs) and increased content of lipid species composed of saturated fatty acids (SFAs) and some breakdown products (e.g., choline, glycerophosphocholine). Furthermore, significant impairments were also observed in biological pathways related to energy metabolism, neurotransmitter levels and fatty acid homeostasis. To complement this study, a FI-APPI-MS approach was subsequently applied to focus on the less polar metabolome, non-readily detectable by ESI-based metabolomics [22]. Increased serum levels of diacylglycerols and ceramides were detected in AD patients, indicative of up-regulated degradation of membrane phospholipids and sphingolipids by the action of phospholipases and sphingomyelinases, in line with results from DIMS analysis. Due to the central role that lipid dyshomeostasis seems to play in AD pathogenesis, serum samples from the same cohort of AD patients were subjected to DIMS-based lipidomics using a modification of the Bligh-Dyer extraction method [23]. Again, a reduced content of PUFA-containing phospholipids and increased levels of diacylglycerols were observed, corroborating previous hypotheses. Furthermore, changes in other low molecular weight metabolites also evidenced severe impairments in the homeostasis of various neurotransmitter systems, nitrogen metabolism and oxidative stress. Taking into account this evidence about the major role that phospholipids play in AD etiology, a metabolomic multiplatform based on the combination of DIMS and LC-MS, this later coupled to both molecular (ESI) and elemental (inductively coupled plasma, ICP) mass spectrometry was employed to get a deeper understanding of the AD-associated phospholipidome [24]. Thus, results evidenced that multiple factors are involved in this abnormal phospholipid homeostasis, including the imbalance of PUFA/SFA contained in their structure, the up-regulation of phospholipases, the implication of oxidative stress and peroxysomal malfunctioning, among others. Complementarily, González-Domínguez et al. also employed the DIMS and FI-APPI-MS approaches previously described to investigate the AD-like pathology in various transgenic mice models compared with wild type (WT) littermates. The analysis of serum samples from APP × PS1 mice revealed analogous metabolomic disturbances to those detected in previous studies with human cohorts, demonstrating the potential of these transgenic animals to model AD [25]. Additionally, DIMS-based fingerprinting has also been applied to the APP × PS1 × IL4-KO transgenic model with the aim of investigating the role of inflammation induced by means of interleukin-4 depletion in AD pathology [26]. Alterations in serum levels of eicosanoids, amino acids and related compounds, and metabolites involved in the urea cycle demonstrated that depletion of interleukin-4 exacerbates AD pathology in this transgenic line. It should be noted that all these results obtained by DMS analysis were subsequently validated by applying various orthogonal metabolomic techniques, including LC-MS, GC-MS and CE-MS [45,46,47,48], thus demonstrating the potential of MS-fingerprinting approaches to carry out fast and accurate screening of complex metabolic networks.

Other published studies on DMS-based metabolomics have focused on the characterization of metabolic impairments observed in brain from various transgenic mice models, a tissue of great interest in AD research, since it enables the in situ investigation of neuropathological processes related to this neurodegenerative disorder. Lin et al. applied an optimized DIMS platform to look for characteristic metabolic impairments in the hippocampus [27] and cerebellum [28] of the CRND8 mouse model. Major findings were observed with regard to an abnormal metabolism of amino acids and nucleotides, as well as the over-production of eicosanoids. In this vein, DIMS-based analysis of various brain regions from the APP × PS1 mouse model (i.e., hippocampus, cortex, cerebellum, striatum, and olfactory bulbs) evidenced that hippocampus and cortex are the most perturbed regions in AD pathology [29]. Similarly to previous studies, significant differences were observed in levels of phospholipids, acyl-carnitines, fatty acids, nucleotides, amino acids and many other metabolites, results which were then confirmed by LC/GC-MS metabolomic analysis [49]. Recently, Wood et al. also employed a lipidomic approach based on DIMS to define potential biomarkers with the aim of distinguishing healthy controls (HC) from MCI and AD patients [30]. They analyzed frontal cortex grey, white matter and cerebrospinal fluid (CSF), and detected abnormal levels of various lipid classes (e.g., plasmalogens, phosphatidylethanolamines, diglycerides), in agreement with previous studies. Alternatively, other peripheral organs from the APP × PS1 model have also been investigated to assess the possible systemic nature of AD, including the liver, kidneys, spleen and thymus [31]. In this work, authors found significant impairments associated with oxidative stress, lipid dyshomeostasis and imbalances in energy metabolism, among other processes, results which were subsequently validated by using a metabolomic multiplatform based on the combination of LC and GC coupled to MS [50,51]. Moreover, urine can also serve as a valid biological sample to study metabolomic perturbations associated with AD by using DIMS-based approaches, as demonstrated by González-Domínguez et al. [32]. For this purpose, various sample preparation methods and normalization strategies were tested, evidencing that ten-fold dilution of urine prior to MS-fingerprinting and subsequent statistical data normalization is enough to minimize ion suppression and to correct the inherent inter-individual variability of this matrix, respectively.

From a targeted perspective, the MDMS-SL platform optimized by Han et al. is a very interesting alternative for the comprehensive investigation of lipidomic alterations associated with AD, in samples coming from both human and animal models. The application of this tool to blood and brain samples showed significant changes in the content of plasmalogens [33], sulfatides [34,35,36], ceramides [34,37] and sphingomyelins [37], thus corroborating the pivotal role of lipid metabolism in the pathogenesis of AD. On the other hand, other authors proposed the use of AbsoluteIDQ^TM^ kits to analyze blood, brain and CSF samples from AD and MCI patients, observing major changes in the content of phospholipids and acyl-carnitines [39,40,41,42,43,44]. However, it should be noted that this tool presents a great drawback in the form of its low metabolome coverage.

## 4. Conclusions

Metabolomic approaches based on DMS analysis have been gaining great importance in recent years because of their high-throughput screening potential, reduced analysis time and wide metabolome coverage. In particular, these platforms have been widely applied for characterizing multifactorial disorders such as Alzheimer’s disease, with the aim of elucidating the pathological mechanisms underlying disease development and progression and discovering potential diagnostic biomarkers. The analysis of multiple biological samples, including serum/plasma, urine, brain (hippocampus, cortex, cerebellum, etc.), cerebrospinal fluid and other organs (liver, kidney, spleen, thymus), has enabled obtaining a comprehensive snapshot of the major metabolic hallmarks associated with this neurodegenerative disorder, such as impairments in the homeostasis of membrane lipids, oxidative stress, inflammatory processes, imbalance in energy metabolism and neurotransmitter metabolism, among many others.

## Figures and Tables

**Table 1:** Summary of DMS-based metabolomics studies on Alzheimer’s disease.

Cohort	Sample	Results	Ref.
AD (N = 22) HC (N = 18)	serum	imbalances in the PUFA/SFA composition of phospholipids; impairments in energy metabolism, neurotransmission, fatty acid homeostasis; hyperlipidemia	[20]
AD (N = 22) HC (N = 18)	serum	imbalances in the PUFA/SFA composition of phospholipids	[21]
AD (N = 30) HC (N = 30)	serum	up-regulated degradation of membrane phospholipids and sphingolipids (↑ diacylglycerols, ceramides); impairments in neurotransmission	[22]
AD (N = 22) HC (N = 18)	serum	impairments in membrane phospholipids (↓ PUFA, ↑diacylglycerols), homeostasis of neurotransmitter systems, nitrogen metabolism and oxidative stress	[23]
AD (N = 19) HC (N = 17)	serum	abnormal phospholipid homeostasis (imbalance of PUFA/SFA, over-activation of phospholipases, oxidative stress, peroxysomal dysfunction)	[24]
APP × PS1 (N = 30) WT (N = 30)	serum	impairments in phospholipid homeostasis, energy-related metabolism, oxidative stress, hyperlipidemia, hyperammonemia	[25]
APP × PS1 × IL4-KO (N = 7) APP × PS1 (N = 7) WT (N = 7)	serum	up-regulated production of eicosanoids, altered metabolism of amino acids and urea cycle	[26]
CRND8 (N = 6) WT (N = 6)	hippocampus	altered metabolism of arachidonic acid, carbohydrates and nucleotides	[27]
CRND8 (N = 6) WT (N = 6)	cerebellum	up-regulated production of eicosanoids; altered metabolism of amino acids and nucleotides	[28]
APP × PS1 (N = 30) WT (N = 30)	hippocampus, cortex, cerebellum, olfactory bulb	disturbances in the homeostasis of phospholipids, acyl-carnitines, fatty acids, nucleotides, amino acids, steroids, energy-related metabolites	[29]
AD young (N = 17) AD old (N = 17) MCI (N = 19) HC young (N = 20) HC old (N = 8)	CSF, frontal cortex grey and white matter	abnormal lipid homeostasis (plasmalogens, phosphatidylethanolamines, diacylglycerols)	[30]
APP × PS1 (N = 30) WT (N = 30)	liver, kidney, spleen, thymus	oxidative stress, lipid dyshomeostasis, imbalances in energy metabolism, homeostasis of amino acids and nucleotides	[31]
APP × PS1 (N = 10) WT (N = 10)	urine	unidentified discriminant signals	[32]
AD (N = 24) HC (N = 6) APPV_717F_, APP_sw_, WT	superior frontal cortex, superior temporal cortex, inferior parietal cortex, cerebellum	plasmalogen deficiency	[33]
AD (N = 17), HC (N = 5)	middle frontal gyrus, superior temporal gyrus, inferior parietal lobule, hippocampus, subiculum, entorhinal cortex	sulfatide deficiency	[34]
APP_V717F_, APP_sw_, WT	cortex, cerebellum	sulfatide deficiency	[35]
AD (N = 6) HC (N = 8)	superior frontal gyrus	sulfatide deficiency	[36]
AD (N = 26) HC (N = 26)	plasma	altered sphingolipidome	[37]
AD (N = 93) HC (N = 99)	serum	authors failed to replicate the 10-metabolite panel described by Mapstone et al. [38]	[39]
MCI (N = 28) HC (N = 73)	plasma	discovery of a panel of 24 metabolites mainly phospholipids and acyl-carnitines)	[40]
AD (N = 143) MCI (N = 145) HC (N = 153)	plasma	impairments in phospholipid homeostasis	[41]
AD (N = 53) MCI (N = 33) HC (N = 35)	plasma	impairments in phospholipid homeostasis	[42]
AD, MCI, HC	brain, serum	impairments in the homeostasis of phospholipids and sphingolipids	[43]
APP × PS1 (N = 9) WT (N = 9)	brain, plasma	impairments in the homeostasis of phospholipids, acyl-carnitines, amino acids and polyamines	[44]
